# The relationship between duration of infertility and clinical outcomes of intrauterine insemination for younger women: a retrospective clinical study

**DOI:** 10.1186/s12884-024-06398-y

**Published:** 2024-03-14

**Authors:** Chenyang Huang, Qingqing Shi, Jun Xing, Yuan Yan, Xiaoyue Shen, Huizhi Shan, Haixiang Sun, Jie Mei

**Affiliations:** Center for Reproductive Medicine and Obstetrics and Gynecology, Drum Tower Clinic Medical College of Nanjing Medical University, Nanjing, 210008 China

**Keywords:** Duration of infertility, Intrauterine insemination, Clinical pregnancy rate, Live birth rate, Female age

## Abstract

**Background:**

The objective of this research was to elucidate the association between the length of infertility and the outcomes of intrauterine insemination (IUI) in women of varying ages - a topic that has been the subject of investigation for numerous years, yet lacks a definitive consensus.

**Methods:**

A retrospective cohort investigation involving 5268 IUI cycles was undertaken at the Reproductive Medicine Center of Nanjing Drum Tower Hospital from 2016 to 2022. Utilizing the smooth fitting curve along with threshold and saturation effect analysis, the correlation between infertility duration and IUI clinical pregnancy rates was discerned. Moreover, patients were bifurcated into two cohorts based on their respective infertility durations. A secondary examination was also performed employing propensity-score matching to mitigate the impact of confounding variables. Subsequent threshold and saturation effect analysis was carried out across various subgroups, segmented on the basis of age differentiation.

**Results:**

When the duration of infertility was more than 5 years, the clinical pregnancy rate decreased with the increase of infertility duration (aOR: 0.894, 95%CI: 0.817–0.991, *p* = 0.043). The multivariate regression analysis suggested that longer duration of infertility (≥ 5 years) was significantly correlated with the lower clinical pregnancy rate (aOR: 0.782, 95% CI: 0.643–0.950, *p* = 0.01). After the propensity-score matching, the clinical pregnancy rate of women with longer infertility duration were also higher. When the duration of infertility was more than 5 years, the clinical pregnancy rate of women younger than 35 years old decreased with the increase of infertility duration (aOR: 0.906, 95%CI: 0.800–0.998, *p* = 0.043).

**Conclusions:**

The clinical pregnancy rate and live birth rate of IUI in young women (< 35 years old) who have been infertile for more than 5 years significantly decrease with the prolongation of infertility time. Therefore, for young women who have been infertile for more than 5 years, IUI may not be the best choice.

**Supplementary Information:**

The online version contains supplementary material available at 10.1186/s12884-024-06398-y.

## Background

Infertility represents a significant global health issue, with a projected prevalence rate ranging from 10 to 15%. It is estimated that one in every seven couples grapples with an unfulfilled desire to conceive for a duration exceeding one year [1, 2]. Intrauterine insemination (IUI) has always been a first line assisted reproductive therapy (ART) for infertility due to its ease of management, low cost, low incidence of complications and closer approach to natural pregnancy [3, 4]. The current guidelines point out that IUI should be a first-line treatment for unexplained infertile women with appropriate age or ovarian reserve function [5]. However, the clinical pregnancy rate and live birth rate of IUI is low, which was reported a live birth rate of 10% [6] for IUI cycles. Patients often require multiple cycles of artificial insemination to enhance the cumulative rates of clinical pregnancy and live birth. This necessity may precipitate depression and emotional distress in couples, potentially impacting subsequent ART outcomes [7–10]. Therefore, early identification of the possibility of pregnancy after IUI is beneficial for better medical consultation and treatment planning [11]. There are numerous factors affecting the outcomes of IUI cycles described previously, which include the female age, the type of infertility, the infertility duration, the number of IUI cycles, the initial dosage of gonadotropin (Gn), the number of dominant follicles, endometrial thickness, and the sperm parameters [1, 4, 12]. There have been some reports [13, 14] regarding the duration of infertility as a predictive factor for pregnancy outcomes after IUI treatment. Certain research studies propose that extended periods of infertility are correlated with a decreased pregnancy rate following IUI [1, 15]. However, Tay et al. found no relationship between the duration of infertility and pregnancy outcomes [16]. These studies have not achieved a unanimous agreement. Consequently, we undertook a retrospective analysis of IUI cycles at our reproductive medicine centre spanning the years 2016 to 2022. The objective was to investigate the correlation between the duration of infertility and clinical pregnancy outcomes in IUI cycles among women of varying age groups.

## Methods

### Patient population

From January 2016 to December 2022, there were 5268 IUI cycles conducted in our retrospective study at the reproductive medicine centre of Nanjing Drum Tower Hospital. All patients underwent preliminary examinations for infertility, such as fallopian tube patency test, gynecological pelvic ultrasound, basal female sex hormones, and male semen examination. Women who meet the IUI treatment criteria (no abnormal findings in the evaluation of semen analysis, uterine and fallopian tube patency, and ovulation function) were included in this study. Exclusion criteria: (1) patients with previous IUI cycles in other hospital; (2) patients with abnormal chromosomal karyotype; (3) patients with lesions of the uterine cavity and endometrium, endometriosis or adenomyosis; (4) the loss of follow-up; (5) the missing data records; (6) the canceled IUI cycles due to various reasons (such as early ovulation, thin endometrium, poor semen quality, personal factors, etc.). All patients have signed informed consent forms. At the same time, this study was approved by the ethics committee of Nanjing drum tower hospital, with ethical batch number 2021-384-01.

### Procedures

IUI cycle protocol: A natural cycle (NC) is implemented for women who have regular menstrual cycles (prior normal ovulation) and for cases of male factor infertility. Follicular ultrasound monitoring is conducted from the 10th to the 12th day of the menstrual cycle. The timing for the administration of human chorionic gonadotropin (hCG, Chorionic Gonadotropin for Injection, Livzon Pharm, China, 5000–10,000 IU) is determined by the combination of serum oestrogen (E2) levels, luteinizing hormone (LH) levels, and the diameter of the dominant follicle. IUI is performed once on the first day post hCG injection. If ultrasound evaluation does not indicate ovulation, IUI is repeated on the second day post hCG injection. However, if ultrasound demonstrates ovulation on the first day post hCG injection, IUI is only performed once. An ovulation induction (OI) cycle is implemented for patients exhibiting irregular menstrual cycles (characterized by disorders in follicular development or ovulation) or for those who have been unsuccessful in achieving pregnancy following multiple NC-IUIs. Patients typically commence ovarian stimulation between the third to fifth day of their menstrual cycle. Medications such as clomiphene citrate (CC, Codal Synto Co., Ltd., Limassol, Cyprus), letrozole (LE; Jiangsu Hengrui Pharmaceutical Co., Ltd., Lianyungang, China), and human menopausal gonadotropin (hMG; Livzon Pharmaceutical, Zhuhai, China) are utilized in the OI cycles. The dosage of these drugs is determined based on the patient’s BMI, AFC, and previous ovarian responsiveness. The timing for the administration of hCG is determined by the combination of serum E_2_ levels, LH levels, and the diameter of the dominant follicle. IUI is performed once on the first day post hCG injection. If ultrasound evaluation does not indicate ovulation, IUI is repeated on the second day post hCG injection. However, if ultrasound demonstrates ovulation on the first day post hCG injection, IUI is only performed once.

IUI: The male partner is advised to abstain from sexual activity for a duration of 3 to 7 days prior to semen collection. The semen is then processed using the double wash gradient method, resulting in a final volume of 0.8 mL post-treatment. The processed sperm suspension is then gently introduced into the uterine cavity utilizing a catheter specifically designed for IUI procedures.

Luteal support: After IUI, patients were given oral dydrogesterone (Duphaston, Dutch Abbott Biologics, 10 mg, b.i.d.) starting from the day of ovulation. Serum β-human chorionic gonadotropin (β-hCG) levels were measured at 16–18 days after IUI. The medication was discontinued for patients with negative β-hCG, while medication was continued until 10 weeks of pregnancy for patients with persistent pregnancy.

Follow-up: If the serum β-hCG was greater than 20 U/L, it was diagnosed as biochemical pregnancy. On the 35th day after IUI, ultrasound examination revealed one or more gestational sacs, which were diagnosed as the clinical pregnancy. Follow up until delivery was continued, and the patient’s final pregnancy outcome was recorded. The spontaneous abortion occurring before 12 weeks of pregnancy is defined as the early miscarriage. The live birth was defined as the delivery of a living newborn after the 28th gestational week.

Primary outcome: Clinical pregnancy rate, defined as the ratio of the number of clinical pregnancy cycles to the total number of IUI cycles.

Secondary outcomes: Live birth rate, calculated as the ratio of the live birth cycle number to the number of embryo transfer cycles. Early miscarriage rate, calculated as the ratio of the early miscarriage cycle number to the number of clinical pregnancy cycles.

### Statistical analysis

Like our previous statistical analysis reported [17], the threshold and saturation effect analyses were conducted to explore the cut-off value of the infertility duration and the IUI cycles were divided into groups A and B according to different duration of infertility. A univariate analysis was employed to identify potential influencing factors of clinical pregnancy outcomes in IUI cycles. And then a multivariable logistic-regression model was conducted for further analysis including the identified factors [18]. In addition, the propensity-score method was used to reduce the effects of confounding. Matching was performed with the use of a 1:1 matching protocol without replacement, with a caliper width equal to 0.01 of the standard deviation of the logit of the propensity score [18]. Furthermore, subgroup analysis based on age stratification was conducted to elucidate the impact of different infertility duration on clinical pregnancy outcomes. A t-test was used for the normally distributed variables and the Mann-Whitney U test was used for the the non-normally distributed variables. Furthermore, the chi-squared test was employed for the categorical variables. All variables are presented as the mean ± standard deviation (SD). The two-sided a level was set at 0.05. All statistical analyses were performed using EmpowerStats (www.empowerstats.com, X&Y solutions, Inc. Boston MA) and R software version 3.6.0 (http://www.r-project.org) [17].

## Results

### Relationship between infertility duration and the clinical pregnancy rate

**Fig. 1:**
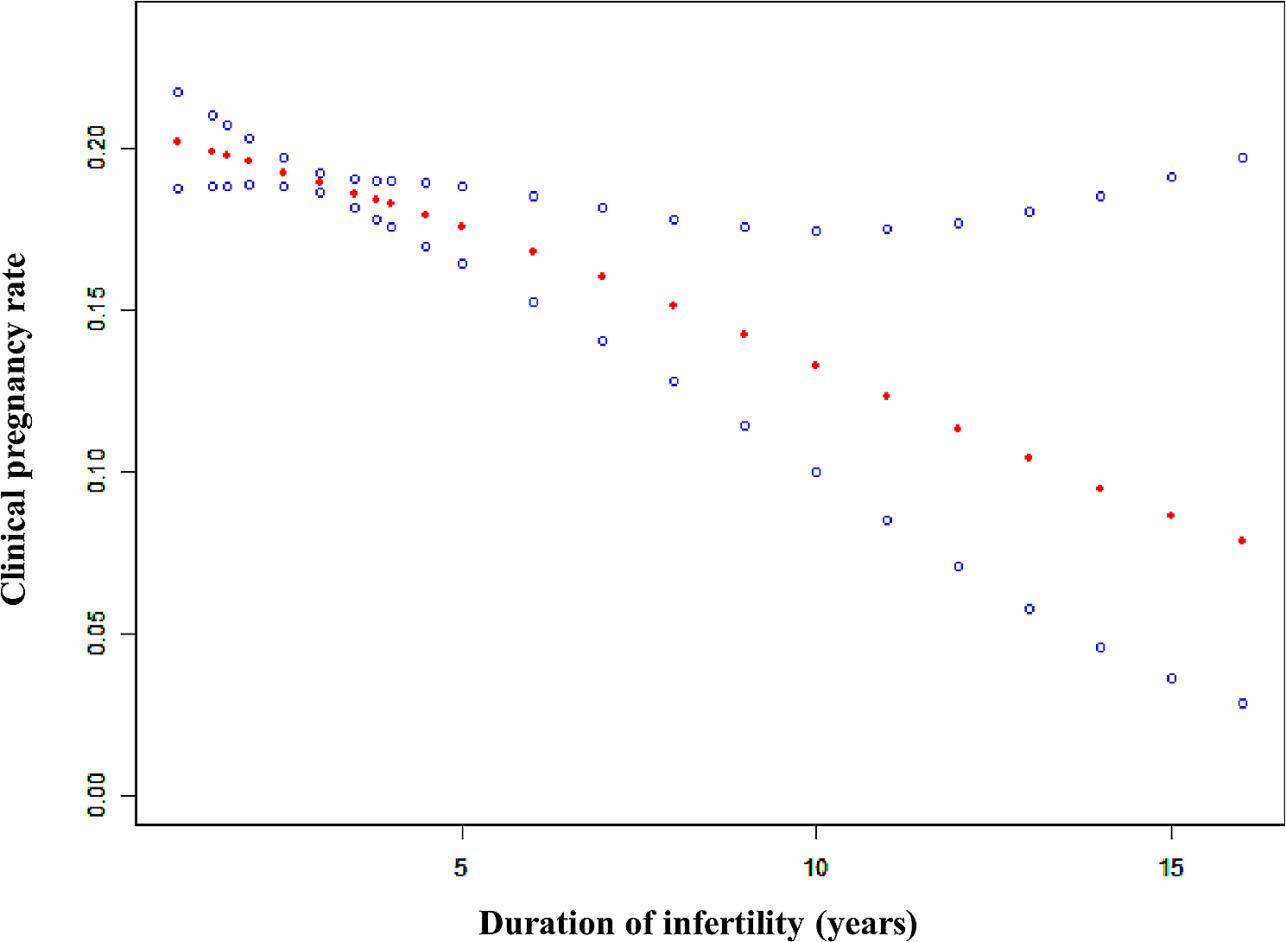
A smooth fitting curve analysis between duration of infertility and clinical pregnancy rates of IUI cycles. The clinical pregnancy rate of the IUI patients decreased obviously as the infertility duration gradually increased

The clinical pregnancy rate decreased obviously as the infertility duration gradually increased (Fig. 1). To clarify the cut-off value of duration of infertility, the threshold effect analysis revealed a curvilinear relationship between the infertility duration and the clinical pregnancy rate (Table 1, logarithmic likelihood ratio = 0.024). As the duration of infertility was more than 5 years, the clinical pregnancy rate declined with the prolonged infertility duration (Table 1, aOR: 0.894, 95%CI: 0.817–0.991, *p* = 0.043).

**Table 1:** Threshold effect analysis of infertility duration (years) on the CPR

Outcome	Clinical pregnancy		
**Model I (linear)**	**aOR**	**95% CI**	**p value**
**Linear effect**	0.950	(0.910, 0.990)	0.057
**Model II (polyline)**	**aOR**	**95% CI**	**p value**
**Predicted threshold (K, infertility duration, years)**	5.0		
**Effect 1 (< K)**	0.978	(0.916, 1.026)	0.392
**Effect 2 (> K)**	0.894	(0.817, 0.991)	0.043
**variability of effectiveness**	0.926	(0.808, 1.061)	0.251
**Logarithmic likelihood ratio test**	0.024		

CPR: clinical pregnancy rate; aOR: adjusted odds ratio; CI: confidence interval; K: predicted threshold

**Adjust for**: female age, male age, BMI, Baseline FSH, AFC, number of cycles, protocol, endometrial thickness and number of progressive motility spermatozoa after treatment

### Characteristics of these 2 groups according to different infertility duration

All IUI cycles were divided into two groups based on different duration of infertility: Group A - duration < 5 years and Group B - duration ≥ 5 years. Female age, female body mass index (BMI) and male age were lower in group A, and antral follicle count (AFC) was larger in group B. There were no significant differences in the type of infertility, baseline follicle-stimulating hormone (FSH), number of previous IUI cycles, protocols of IUI cycles, number of progressive motility spermatozoa after treatment, endometrial thickness, and number of IUI between the two groups. The clinical pregnancy rate was higher in group A, similar to the live birth rate. In addition, there was no difference of the early miscarriage rate between these 2 groups. A better clinical outcome could be detected in IUI cycles of patients with a lower duration of infertility (< 5 years).

### Multivariate regression analysis and propensity-score matching for these 2 groups

A univariate analysis was conducted to identify potential confounding factors which might have effect on the clinical outcomes (Table S1). Several factors (female age, male age, BMI, FSH, AFC, number of cycles, protocol, endometrial thickness, and number of progressive motility spermatozoa after treatment) were identified as confounding variables and selected as adjusted variables in the multivariate regression analysis. Longer infertility duration (≥ 5 years) was significantly correlated with the lower clinical pregnancy rate (Table 2, aOR = 0.782, 95% CI: 0.643–0.950, *p* = 0.01). There were significant differences in some baseline characteristics between the two groups (Table 3). To reduce the impact of these confounding factors, we used propensity-score matching to screen patients with similar baseline characteristics. The clinical pregnancy and live birth rates of IUI cycles in group A were higher than those in group B significantly (Table 4).

**Table 2:** Multivariate analysis for infertility duration in IUI cycles involved in the clinical pregnancy rate

Infertility duration groups	Adjusted OR	95% CI	p value
**Group A (< 5 years)**	1.000	1.000	
**Group B (≥ 5 years)**	0.782	0.643–0.950	0.01

**Adjust for**: female age, male age, BMI, Baseline FSH, AFC, number of cycles, protocol, endometrial thickness and number of progressive motility spermatozoa after treatment

**Table 3:** Characteristics of IUI cycles according to different infertility duration

	Group A (duration < 5 years) (*n* = 4264)	Group B (duration ≥ 5 years) (*n* = 1004)	P value
**Female age, years**	29.1 ± 3.5	31.1 ± 3.2	< 0.01
**Male age, years**	30.4 ± 4.2	32.8 ± 3.9	< 0.01
**BMI, kg/m** ^**2**^	23.0 ± 3.5	23.3 ± 3.7	< 0.01
**Baseline FSH, IU/L**	7.2 ± 2.0	7.2 ± 1.9	0.82
**AFC, n**	19.3 ± 7.8	18.1 ± 7.5	< 0.01
**Type of infertility**			0.17
**Primary infertility, n** **Secondary infertility, n**	2973 (69.7%) 1291 (30.3%)	722 (71.9%) 282 (28.1%)	
**IUI cycles, n**	1.6 ± 0.8	1.6 ± 0.7	0.35
**Protocol**			0.74
**Natural cycle**	623 (14.6%)	157 (15.6%)	
**CC for OI cycle**	1017 (23.9%)	240 (23.9%)	
**LE for OI cycle**	2316 (54.3%)	542 (54.0%)	
**HMG for OI cycle**	308 (7.2%)	65 (6.5%)	
**Endometrial thickness, mm**	10.1 ± 2.0	10.1 ± 1.9	0.77
**Number of IUI, n**			0.36
**1** **2**	748 (17.5%) 3516 (82.5%)	164 (16.3%) 840 (83.7%)	
**Number of progressive motility spermatozoa after treatment, million**	17.4 ± 11.3	16.3 ± 10.9	0.33
**Clinical pregnancy rate**	19.6% (834)	15.0% (151)	< 0.01
**Early miscarriage rate**	15.3% (128)	18.5% (28)	0.39
**Live birth rate**	16.1% (688)	12.0% (120)	< 0.01

**Table 4:** Characteristics of IUI cycles according to different infertility duration after Propensity-Score Matching

	Group A (duration < 5 years) (*n* = 1004)	Group B (duration ≥ 5 years) (*n* = 1004)	P value
**Female age, years**	30.9 ± 3.4	31.1 ± 3.2	0.22
**Male age, years**	32.3 ± 4.4	32.8 ± 3.9	0.24
**BMI, kg/m** ^**2**^	23.1 ± 3.6	23.3 ± 3.7	0.35
**Baseline FSH, IU/L**	7.2 ± 2.1	7.2 ± 1.9	0.42
**AFC, n**	18.2 ± 7.2	18.1 ± 7.5	0.92
**Type of infertility**			0.96
**Primary infertility, n** **Secondary infertility, n**	718 (71.7%) 284 (28.3%)	722 (71.9%) 282 (28.1%)	
**IUI cycles, n**	1.6 ± 0.7	1.6 ± 0.7	0.97
**Protocol**			0.68
**Natural cycle**	141 (14.1%)	157 (15.6%)	
**CC for OI cycle**	254 (25.3%)	240 (23.9%)	
**LE for OI cycle**	550 (54.7%)	542 (54.0%)	
**HMG for OI cycle**	59 (5.9%)	65 (6.5%)	
**Endometrial thickness, mm**	10.1 ± 2.0	10.1 ± 1.9	0.82
**Number of IUI, n**			0.34
**1** **2**	180 (18.0%) 824 (82.0%)	164 (16.3%) 840 (83.7%)	
**Number of progressive motility spermatozoa after treatment, million**	16.7 ± 11.5	16.3 ± 10.9	0.32
**Clinical pregnancy rate**	19.6% (197)	15.0% (151)	< 0.01
**Early miscarriage rate**	16.8% (33)	18.5% (28)	0.77
**Live birth rate**	15.5% (156)	12.0% (120)	0.02

### Relationship between infertility duration and the clinical outcome of younger women

Older patients may experience longer periods of infertility. Therefore, there may be different cut-off values for infertility duration for patients of different ages. We conducted further subgroup analysis and divided all patients into young (< 35 years old) and elderly (≥ 35 years old) groups. There was a curvilinear relationship between infertility duration and the clinical pregnancy rate of younger women in a threshold effect analysis (Table S2, logarithmic likelihood ratio = 0.039). As the infertility duration was more than 5 years, the clinical pregnancy rate of younger patients (< 35 years old) decreased with the increase of infertility duration (Table S2, aOR: 0.906, 95%CI: 0.800-0.998, *p* = 0.043). And 5 years is not the cut-off value applicable to the elderly group.

## Discussion

The clinical pregnancy outcomes of IUI are influenced by various factors, such as the basic conditions of the couple, treatment protocol, timing of insemination, and sperm quality after treatment [19–22]. We posit that the fundamental characteristics of a patient serve as the initial criteria for making clinical decisions. Hence, we conducted a retrospective analysis to investigate the correlation between the duration of infertility and the clinical pregnancy outcomes following IUI in patients of varying ages. Our study revealed that for patients who have been infertile for more than 5 years, both the clinical pregnancy rate and live birth rate following IUI progressively decrease as the duration of infertility extends.

The correlation between the duration of infertility and the clinical outcomes of IUI has been a topic of extensive debate. Numerous researchers posit that the length of infertility serves as a significant predictor for successful pregnancy, with the assertion that patients experiencing prolonged periods of infertility exhibit significantly reduced pregnancy rates [23, 24]. Previous studies have shown that the clinical pregnancy rate decreases with the duration of infertility in patients [1, 15, 25]. There were also studies suggested that approximately 80% of pregnancies are achieved less than 4 years after attempting. Although it is difficult to determine a threshold value of infertility duration, less than 4–5 years of infertility should be a appropriate time to try IUI [26]. This is similar to our study, as our results suggest that patients who have been infertile for more than 5 years have significantly reduced clinical pregnancy rates and live birth rates of IUI cycles. On the contrary, some studies suggested that there was no correlation between the duration of infertility and IUI clinical outcomes [16, 27]. The major reason for this difference may be the difference in basic characteristics of the patient population. One of the studies was conducted on polycystic ovarian syndrome (PCOS) patients, and the results showed no significant difference in the duration of infertility between the pregnant and non pregnant groups [28]. However, PCOS often results in extended periods of infertility and missed opportunities for conception due to aberrant ovulation or anovulation. Thus, the impact of the duration of infertility on the clinical pregnancy outcomes in these patients is ostensibly negligible.

Female age is one of the most important factors for pregnancy, and it is an important predictor of ART success, which have been reported in most studies [1, 4, 29, 30]. It is generally believed that advanced age will reduce women’s fertility, and the number of oocytes consumed rapidly in women over 35 years old. The storage of metabolites can alter the ovarian environment, such as DNA mutations and telomere shortening, thereby reducing the quality of oocytes [31]. In addition, advanced age is often accompanied by a decrease in endometrial receptivity [32, 33]. Many studies have shown a significant decrease in the clinical pregnancy rate of IUI of women with the age over 37 years old [34–36]. In addition to the adverse impact of advanced age on the clinical pregnancy rate of IUI, elderly patients typically experience longer periods of infertility. Therefore, we conducted an age stratified analysis of the patients enrolled in this study. Our study found that when patients are younger than 35 years old, the cut-off value for infertility duration still exists. When young patients have been infertile for more than 5 years, their clinical pregnancy rate and live birth rate of IUI significantly decrease. When the patient is over 35 years old, the clinical pregnancy rate and live birth rate are much lower than those of young patients. At the same time, the cut-off value for infertility years is no longer applicable to elderly patients. Therefore, for young patients (< 35 years old), if the infertility period exceeds 5 years, IUI may not be the optimal choice. For elderly patients (≥ 35 years old), regardless of the length of infertility, it is necessary to be more cautious when choosing IUI treatment.

This study is not without limitations. Due to the relatively small sample size of older patients, we were unable to derive an exact cut-off value for their duration of infertility. Our data system’s constraints made it challenging to directly obtain comprehensive data of patients in the IUI cycles, such as the number and size of leading follicles. Consequently, we were unable to conduct a thorough analysis of potential influence from other variables and subsequent in vitro fertilization (IVF) cycles for patients unsuccessful in achieving pregnancy through IUI. The primary limitation of this study is its retrospective design. While these results can be cautiously applied in a real-world setting, further elucidation of the effects of female age and duration of infertility on the clinical pregnancy outcomes of IUI cycles necessitates high-quality, large-scale randomized controlled trials. This will enable more accurate selection of IUI treatment, thereby avoiding unnecessary time and financial expenditure. In addition, besides the basic patients’ data, there are some interventions that can affect the clinical pregnancy outcome of IUI. For example, studies suggested that endometrial scratching in the previous cycle (luteal phase) or in the IUI cycle (follicular phase) can improve the clinical pregnancy outcome [37, 38]. At the same time, it does not increase the risk of multiple pregnancies, miscarriages, or ectopic pregnancies. These are also areas that we need to focus on and explore in order to improve the clinical pregnancy outcome of IUI cycles in the future.

## Conclusion

The clinical pregnancy rate and live birth rate of IUI in young women (< 35 years old) who have been infertile for more than 5 years significantly decrease with the prolongation of infertility time. Therefore, for young women who have been infertile for more than 5 years, IUI may not be the best choice.

### Electronic supplementary material

Below is the link to the electronic supplementary material.

Supplementary Material 1

Supplementary Material 2

Supplementary Material 3

Supplementary Material 4

## Data Availability

The datasets generated and analyzed during the current study are not publicly available due to the special requirements of our hospital and our reproductive medicine center for the disclosure of patients’ clinical data but are available from the corresponding author on reasonable request.

## Abbreviations

IUI: Intrauterine insemination
ART: Assisted reproductive therapy
NC: Natural cycle
E_2_: Oestrogen
LH: Luteinizing hormone
hCG: Human chorionic gonadotropin
OI: Ovulation induction
SD: Standard Deviation
BMI: Body mass index
AFC: Antral follicle count
FSH: Follicle-stimulating hormone
PCOS: Polycystic ovarian syndrome
IVF: In vitro fertilization
