# Supplementary material for: The relationship between duration of infertility and clinical outcomes of intrauterine insemination for younger women: a retrospective clinical study
Source: BMC Pregnancy Childbirth. 2024 Mar 14;24:199. doi: 10.1186/s12884-024-06398-y (PMC10938817; doi:10.1186/s12884-024-06398-y)
Supplement: Supplementary file 1 — Supplementary Material 1 [file 12884_2024_6398_MOESM1_ESM.docx]

**Table S1.** Univariate analysis of total IUI cycles.

| **Total cycles** | **Adjusted OR** | **95% CI** | **p value** |
| --- | --- | --- | --- |
| **Female age** | 0.960 | 0.941-0.980 | <0.01 |
| **Male age** | 0.972 | 0.956-0.989 | <0.01 |
| **BMI** | 1.011 | 1.001-1.021 | 0.02 |
| **Baseline FSH, IU/L** | 0.933 | 0.899-0.968 | <0.01 |
| **AFC** | 1.023 | 1.014-1.032 | <0.01 |
| **Type of infertility** |  |  | 0.19 |
| **Primary infertility**  **Secondary infertility** | 1.000  1.105 | 1.000  0.951-1.283 |  |
| **Infertility duration, years** | 0.939 | 0.904-0.974 | <0.01 |
| **Number of cycles, n** | 0.901 | 0.817-0.993 | 0.03 |
| **Protocol** |  |  |  |
| **Natural cycle** | 1.000 | 1.000 |  |
| **CC for OI cycle** | 1.123 | 0.885-1.424 | 0.34 |
| **LE for OI cycle** | 1.203 | 0.974-1.486 | 0.09 |
| **HMG for OI cycle** | 1.481 | 1.088-1.125 | 0.01 |
| **Endometrial thickness, mm** | 1.052 | 1.017-1.089 | <0.01 |
| **Number of IUI** |  |  | 0.12 |
| **1**  **2** | 1.000  1.160 | 1.000  0.961-1.402 |  |
| **Number of progressive motility spermatozoa, n** | 1.931 | 1.774-1.991 | <0.01 |
