# Supplementary material for: The relationship between duration of infertility and clinical outcomes of intrauterine insemination for younger women: a retrospective clinical study
Source: BMC Pregnancy Childbirth. 2024 Mar 14;24:199. doi: 10.1186/s12884-024-06398-y (PMC10938817; doi:10.1186/s12884-024-06398-y)
Supplement: Supplementary file 2 — Supplementary Material 2 [file 12884_2024_6398_MOESM2_ESM.docx]

**Table S2. Threshold effect analysis of infertility duration (years) on the CPR.**

| **Outcome** | **Clinical pregnancy** | | | | | |
| --- | --- | --- | --- | --- | --- | --- |
| **Different female age** | **< 35 years** | | | **≥ 35 years** | | |
| **Model I (linear)** | **aOR** | **95% CI** | **p value** | **aOR** | **95% CI** | **p value** |
| **Linear effect** | 0.952 | (0.911, 0.995) | 0.077 | 0.931 | (0.852, 1.018) | 0.117 |
| **Model II (polyline)** | **aOR** | **95% CI** | **p value** | **aOR** | **95% CI** | **p value** |
| **Predicted threshold (K, infertility duration, years)** | 5.0 | | | 5.0 | | |
| **Effect 1 (<K)** | 0.969 | (0.912, 1.030) | 0.316 | 1.033 | (0.847, 1.259) | 0.750 |
| **Effect 2 (>K)** | 0.906 | (0.800, 0.998) | 0.043 | 0.850 | (0.703, 1.028) | 0.093 |
| **variability of effectiveness** | 0.935 | (0.799, 1.093) | 0.398 | 0.823 | (0.587, 1.154) | 0.258 |
| **Logarithmic likelihood ratio test** | 0.039 | | | 0.253 | | |

CPR: clinical pregnancy rate; aOR: adjusted odds ratio; CI: confidence interval; K: predicted threshold.

**Adjust for:** female age, male age, BMI, Baseline FSH, AFC, number of cycles, protocol, endometrial thickness and number of progressive motility spermatozoa after treatment
