# Supplementary material for: The relationship between duration of infertility and clinical outcomes of intrauterine insemination for younger women: a retrospective clinical study
Source: BMC Pregnancy Childbirth. 2024 Mar 14;24:199. doi: 10.1186/s12884-024-06398-y (PMC10938817; doi:10.1186/s12884-024-06398-y)
Supplement: Supplementary file 3 — Supplementary Material 3 [file 12884_2024_6398_MOESM3_ESM.docx]

**Table S3.** Characteristics of IUI cycles according to different infertility duration of patients with lower age (< 35 years).

|  | **Group A**  **(duration < 5 years)**  **(n = 3930)** | **Group B**  **(duration ≥ 5 years)**  **(n = 841)** | **P value** |
| --- | --- | --- | --- |
| **Female age, years** | 28.6 ± 2.8 | 30.1 ± 2.5 | <0.01 |
| **Male age, years** | 29.9 ± 3.6 | 31.9 ± 3.3 | <0.01 |
| **BMI, kg/m^2^** | 22.9 ± 3.4 | 23.2 ± 3.7 | 0.06 |
| **Baseline FSH, IU/L** | 7.1 ± 2.0 | 7.1 ± 1.8 | 0.72 |
| **AFC, n** | 19.6 ± 7.8 | 18.2 ± 7.4 | <0.01 |
| **Type of infertility** | | | 0.02 |
| **Primary infertility, n**  **Secondary infertility, n** | 2846 (72.4%)  1084 (27.6%) | 642 (76.3%)  199 (23.7%) |  |
| **IUI cycles, n** | 1.5 ± 0.7 | 1.6 ± 0.7 | 0.62 |
| **Protocol** |  |  | 0.86 |
| **Natural cycle** | 559 (14.2%) | 123 (14.6%) |  |
| **CC for OI cycle** | 923 (23.5%) | 202 (24.0%) |  |
| **LE for OI cycle** | 2155 (54.8%) | 460 (54.7%) |  |
| **HMG for OI cycle** | 293 (7.5%) | 56 (6.7%) |  |
| **Endometrial thickness, mm** | 10.2 ± 2.0 | 10.1 ± 1.9 | 0.82 |
| **Number of IUI, n** |  |  | 0.32 |
| **1**  **2** | 677 (17.2%)  3253 (82.8%) | 133 (15.8%)  708 (84.2%) |  |
| **Number of progressive motility spermatozoa after treatment, million** | 17.5 ± 10.1 | 16.2 ± 10.8 | 0.19 |
| **Clinical pregnancy rate** | 19.9% (781) | 15.6% (131) | <0.01 |
| **Early miscarriage rate** | 14.1% (110) | 16.8% (22) | 0.50 |
| **Live birth rate** | 16.7% (655) | 12.7% (107) | <0.01 |
