# Supplementary material for: The relationship between duration of infertility and clinical outcomes of intrauterine insemination for younger women: a retrospective clinical study
Source: BMC Pregnancy Childbirth. 2024 Mar 14;24:199. doi: 10.1186/s12884-024-06398-y (PMC10938817; doi:10.1186/s12884-024-06398-y)
Supplement: Supplementary file 4 — Supplementary Material 4 [file 12884_2024_6398_MOESM4_ESM.docx]

**Table S4.** Multivariate analysis for infertility duration in IUI cycles involved in the clinical pregnancy rate of younger women (<35 years).

| **Infertility duration groups** | **Adjusted OR** | **95% CI** | **p value** |
| --- | --- | --- | --- |
| **Group A (< 5 years)** | 1.000 | 1.000 |  |
| **Group B (≥ 5 years)** | 0.783 | 0.636-0.964 | 0.02 |

**Adjust for:** male age, BMI, Baseline FSH, AFC, number of cycles, protocol, endometrial thickness and number of progressive motility spermatozoa after treatment
